# Interfacial superconductivity in a bi-collinear antiferromagnetically ordered FeTe monolayer on a topological insulator

**DOI:** 10.1038/ncomms14074

**Published:** 2017-01-17

**Authors:** S. Manna, A. Kamlapure, L. Cornils, T. Hänke, E. M. J. Hedegaard, M. Bremholm, B. B. Iversen, Ph. Hofmann, J. Wiebe, R. Wiesendanger

**Affiliations:** 1 Department of Physics, University of Hamburg, Jungiusstrasse 11, D-20355 Hamburg, Germany; 2 Department of Chemistry and iNANO, Center for Materials Crystallography, Aarhus University, Langelandsgade 140, DK-8000 Aarhus C, Denmark; 3 Department of Physics and Astronomy, Interdisciplinary Nanoscience Center, Aarhus University, DK-8000 Aarhus C, Denmark

## Abstract

The discovery of high-temperature superconductivity in Fe-based compounds triggered numerous investigations on the interplay between superconductivity and magnetism, and on the enhancement of transition temperatures through interface effects. It is widely believed that the emergence of optimal superconductivity is intimately linked to the suppression of long-range antiferromagnetic (AFM) order, although the exact microscopic picture remains elusive because of the lack of atomically resolved data. Here we present spin-polarized scanning tunnelling spectroscopy of ultrathin FeTe_1−*x*_Se_*x*_ (*x*=0, 0.5) films on bulk topological insulators. Surprisingly, we find an energy gap at the Fermi level, indicating superconducting correlations up to *T*_c_∼6 K for one unit cell FeTe grown on Bi_2_Te_3_, in contrast to the non-superconducting bulk FeTe. The gap spatially coexists with bi-collinear AFM order. This finding opens perspectives for theoretical studies of competing orders in Fe-based superconductors and for experimental investigations of exotic phases in superconducting layers on topological insulators.

## Abstract (web summary)

The microscopic picture of how superconductivity is linked to antiferromagnetic order in Fe-based compounds remains elusive. Here, Manna *et al*. report superconducting correlations which spatially coexist with bi-collinear antiferromagnetic order in a one unit cell thin layer of FeTe grown on Bi_2_Te_3_.

Topological insulators (TIs) and interfacial superconductors (SCs) are both topics of intense current interest in modern condensed matter physics^1^,^2^. The combination of both materials is expected to reveal novel physics such as, for example, Majorana Fermions arising in heterostructures of TIs and s-wave SCs by including magnetic fields^3^. Most experimental studies of such heterostructures have concentrated on TI films grown on superconducting bulk substrates^4^. Alternatively, SC/TI heterostructures could be realized by growing superconducting films on top of pristine bulk TIs.

In the past years, studies of Fe-based SCs and their electronic and magnetic properties have been one of the major research fields in condensed matter physics^5^,^6^. The recent finding that the superconducting transition temperature *T*_c_ can be pushed above 100 K in an FeSe unit cell (UC) thin film grown epitaxially on SrTiO_3_ (refs 7, 8, 9) spurred numerous investigations aiming at an exact understanding of how the superconductivity in transition metal mono- and dichalcogenides evolves from bulk to ultrathin films. Bulk FeSe crystals exhibit a *T*_c_ of no higher than 8 K (ref. 10). The significant interfacial enhancement of *T*_c_ in UC FeSe on SrTiO_3_ as well as in similar systems, such as FeSe on BaTiO_3_ (ref. 11) and FeTe_1−*x*_Se_*x*_ on SrTiO_3_^12^, leads to a revival of the idea that tailoring the electron pairing by interfacing a SC with another material can be used to achieve high-temperature SC^1^. Interestingly, SC can even emerge by interfacing two insulators^13^ or a TI (Bi_2_Te_3_) with a non-SC Fe-chalcogenide (FeTe)^14^,^15^. It is therefore of great importance to understand how different substrates affect the electronic, spin-dependent and superconducting properties of single-layer Fe-based SCs. The latter studies^14^,^15^ raised the fundamental question of what happens to the bi-collinear antiferromagnetic (AFM) structure of FeTe (ref. 16) once an interfacial SC state is established in contact with Bi_2_Te_3_. In the work of He *et al*.^14^, it was proposed that the topological surface state may dope the FeTe and suppress the long-range AFM order at the interface, thereby inducing the observed interfacial SC.

By a low-temperature spin-polarized scanning tunnelling spectroscopy (SP-STS) investigation of the correlation between the film structure, the local electronic, superconducting and spin-dependent properties, we show here that, in contrast to this assumption, the energy gap at the Fermi level in UC thin FeTe films epitaxially grown on Bi_2_Te_3_(111) can spatially coexist on the atomic scale with long-range bi-collinear AFM order.

## Results

### Structural properties of FeTe_1−*x*_Se_*x*_ thin films

**Figure 1:**
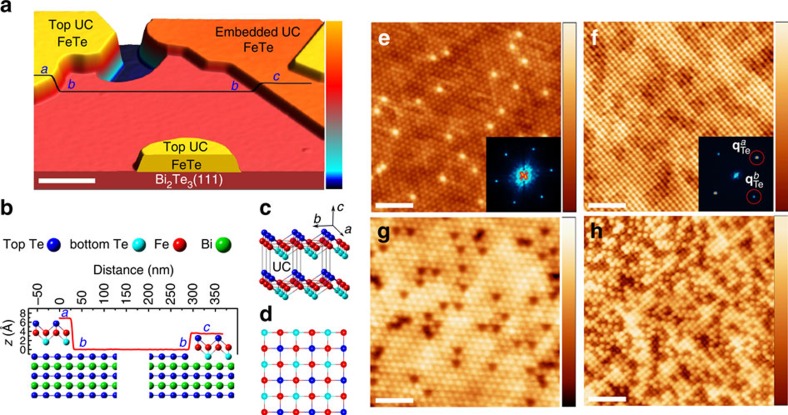
STM topography of FeTe_1−*x*_Se_*x*_ ultrathin films. (**a**) Large-scale constant-current image (*U*=500 mV, *I*_s_=20 pA, white scale bar, 80 nm wide, colour scale from 0 to 1.58 nm apparent height) of one UC FeTe grown on a Bi_2_Te_3_ substrate. (**b**) Measured height profile along the black line (*a*–*b*–c) in **a** revealing embedded (*h*=0.35 nm) and top UC (*h*=0.68 nm) FeTe, consistent with the suggested cross-sectional schematic illustration of the FeTe/Bi_2_Te_3_ heterostructure (not to scale). The schematic crystal structure of an FeTe UC is additionally shown in side- (**c**) and top view (**d**). (**e**) Atomically resolved STM images of a Bi_2_Te_3_ substrate region (*U*=−200 mV, *I*_s_=100 pA, 3 nm lateral scale bar, colour scale from 0 to 105 pm), and (**f**) of a top UC thin FeTe layer (*U*=140 mV, *I*_s_=100 pA, 3 nm lateral scale bar, colour scale from 0 to 84 pm). FFTs (image sizes 0.75 Å^−1^) of the images are displayed in the insets of **e**,**f**, revealing the Bragg peaks associated with the top Te atomic lattice at **q**_Te_^*a*^ and **q**_Te_^*b*^. (**g**) Atomically resolved STM images of the Bi_2_Te_1.8_Se_1.2_ substrate (*U*=50 mV, *I*_s_=1 nA, 2 nm lateral scale bar, colour scale from 0 to 49 pm) and (**h**) of one UC FeTe_0.5_Se_0.5_ layer grown on top (*U*=100 mV, *I*_s_=740 pA, 3 nm lateral scale bar, colour scale from 0 to 159 pm).

We have prepared ultrathin epitaxial FeTe films on Bi_2_Te_3_ single crystals by *in situ* deposition of Fe on the TI substrates under ultrahigh vacuum (UHV) conditions, followed by an *in situ* annealing treatment (see Methods section). Figure 1a depicts a constant-current scanning tunnelling microscopy (STM) topographic image of such a film with FeTe islands of different thicknesses, together with regions of the bare substrate. We distinguish between these different areas based on the measured step heights and differences in the observed atomic-scale surface structures. The line profile displayed in Fig. 1b taken across three different terraces separated by two steps reveals a step height *h* (a–b) of *h*∼0.65±0.05 nm, roughly equal to the *c* axis lattice constant (0.62 nm) of bulk FeTe, while the right step *b*–*c* is considerably smaller (*h*∼0.35±0.05 nm). The smaller step results from an FeTe UC being embedded in the topmost quintuple layer of the substrate, most probably as sketched in Fig. 1b. Figure 1e presents an atomically resolved STM topographic image of the substrate area (Bi_2_Te_3_), which reveals the hexagonal close-packed atomic lattice (see inset Fast Fourier transform (FFT) image) of top Te atoms with a spacing of ∼0.44 nm. A topographic STM image of the top UC layer of FeTe, with an atomic contrast determined by the topmost chalcogenide (Te) layer (Fig. 1f), reveals that the *a* axis of the FeTe is aligned parallel to one of the closed packed directions of the substrate Te atoms. The period of the atomic lattice along the *a* axis direction is ∼0.38 nm, which is consistent with the Te–Te atomic distance on the surface of bulk FeTe (ref. 17). The period along the *b* axis is slightly smaller (*a*/*b*∼1.04), which might be partly attributed to the transition into the orthorhombic/monoclinic phase, known for bulk FeTe^18^, but could be enhanced by uniaxial strain as a result of the heteroepitaxial growth. This holds for the embedded UC FeTe as well as for thicker layers (Supplementary Fig. 1). However, there is no overall periodic height modulation of the UC FeTe layers, and only in some areas of the thinnest islands irregular wrinkles are observed. This indicates a rather weak interaction of the FeTe layer with the substrate, in contrast to what has been observed for FeSe grown on Bi_2_Se_3_ (ref. 19). The Fourier transform of the topographic image (inset of Fig. 1f) clearly shows the peaks associated with the almost square Te atomic lattice, with the spots along the *a* direction (**q**_Te_^*a*^) slightly more intense than those along the *b* direction (**q**_Te_^*b*^; ref. 16). Despite the different lattice symmetries (sixfold for Bi_2_Te_3_ and fourfold for FeTe) with respect to the *c* axis directions and the relatively large lattice mismatch, the heteroepitaxial growth is of very high quality, resulting in an atomically sharp and defect-free interface. In contrast to the surface of bulk FeTe (ref. 16), the surfaces of our ultrathin FeTe films do not show any excess Fe atoms, indicating a stoichiometric FeTe layer.

Although the focus of the present study is on the single layer of FeTe on Bi_2_Te_3_(111), ultrathin FeTe_1−*x*_Se_*x*_ (*x*=0.5) films grown on the ternary TI Bi_2_Te_1.8_Se_1.2_(111) serve as an important reference system^20^. In the corresponding bulk system, FeTe exhibits a bi-collinear AFM order, while superconductivity can be induced and substantially enhanced up to 15 K with partial substitution of Se for Te at *x*=0.5 (ref. 12). It is therefore particularly interesting to investigate how the superconducting state develops from a magnetically ordered state with Se substitution in the thin-film system we studied here.

High-quality, one UC thin FeTe_0.5_Se_0.5_ films (Supplementary Fig. 2) with well-defined stoichiometry were epitaxially grown on Bi_2_Te_1.8_Se_1.2_(111) substrates using a similar preparation method as for the FeTe films. Atomically resolved topographic STM images of the cleaved Bi_2_Te_1.8_Se_1.2_ substrate surface (Fig. 1g) show Se and Te atoms in the topmost layer of the surface appearing with slightly darker and brighter contrast, respectively, with a frequency in accordance with the stoichiometry^21^. A typical topographic STM image of a one UC thin FeTe_0.5_Se_0.5_ film grown on top (Fig. 1h) reveals an ordered square lattice of Se/Te atoms with about half of the atoms appearing brighter (52%, Te sites) while the other half appears darker (48%, Se sites), with a height difference of 65±15 pm, close to the value found for similarly doped FeTe_1−*x*_Se_*x*_ samples^12^,^22^.

### Characterization of superconducting properties

**Figure 2:**
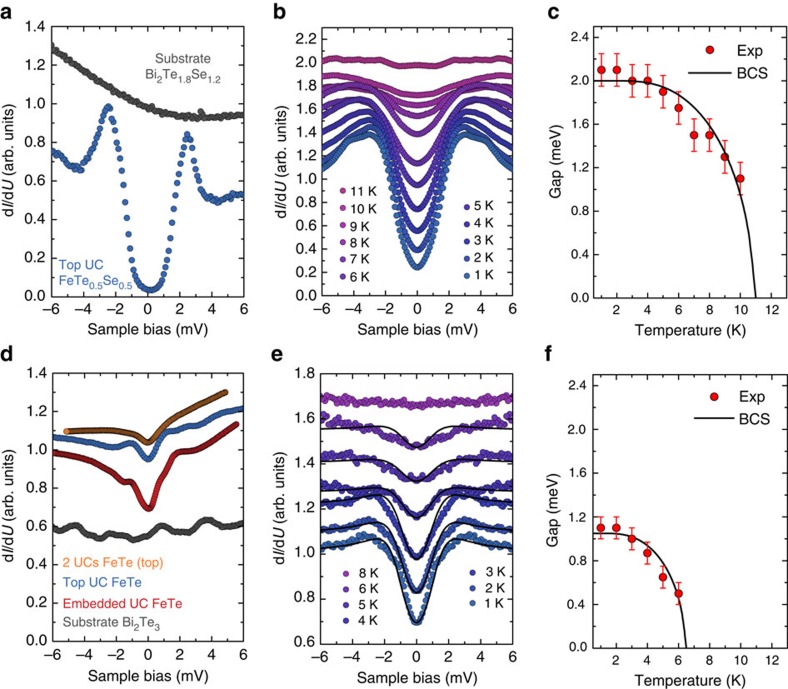
Thickness and temperature dependence of STS on FeTe_1−*x*_Se_*x*_. (**a**) d*I/*d*U* spectra of one UC thin FeTe_0.5_Se_0.5_ and of Bi_2_Te_1.8_Se_1.2_ substrate measured at *T*=1.1 K (*U*=10 mV, *I*_s_=400 pA, *U*_mod_=0.1 mV). (**b**) Temperature evolution of the d*I*/d*U* spectra on a top UC thin FeTe_0.5_Se_0.5_ (*U*=10 mV, *I*_s_=200 pA, *U*_mod_=0.15 mV, spectra vertically offset for clarity). The spectra are spatially averaged along a line of length 1 nm, background-corrected by division through the data measured above *T*_c_ (at *T*=14 K) and finally symmetrized with respect to *U*=0 V. (**c**) Energy gaps Δ of a top UC thin FeTe_0.5_Se_0.5_ as a function of temperature. Markers show the gaps as extracted from Dynes fits (not shown) to the data in **b**, with error bars indicating the maximum range of values that result in an acceptable fitting of the experimental spectra, and the solid line shows the fitting to the BCS gap function. (**d**) Typical spatially averaged d*I/*d*U* spectra measured on ultrathin FeTe films of different thicknesses and on the Bi_2_Te_3_ substrate at *T*=1.1 K. The spectra have been normalized to the d*I*/d*U* value at +5 mV and are vertically offset (substrate: offset −0.4, *U*=10 mV, *I*_s_=170 pA, *U*_mod_=0.2 mV; embedded UC: offset +0.1, *U*=6 mV, *I*_s_=300 pA, *U*_mod_=0.2 mV; top UC: offset +0.2, *U*=10 mV, *I*_s_=600 pA, *U*_mod_=0.25 mV; 2 UCs: offset +0.3, *U*=5 mV, *I*_s_=300 pA, *U*_mod_=0.2 mV). (**e**) Spatially averaged (0.5 nm × 0.5 nm) d*I/*d*U* spectra measured of a top UC thin FeTe layer at temperatures from 1.1 to 8 K (*U*=10 mV, *I*_s_=200 pA, *U*_mod_=0.15 mV, spectra vertically offset for clarity). The spectra are background-corrected by division through the data measured at *T*=8 K, and finally symmetrized with respect to *U*=0 V. The lines are fits to Dynes functions. (**f**) Energy gaps Δ of top UC FeTe, as obtained from the fits in **e**, as a function of temperature. The errors indicate the maximum range of values of Δ in the Dynes fits, which result in an acceptable agreement with the experimental spectra. The solid line represents the temperature variation of Δ as fitted by BCS theory.

To characterize the electronic structure of these films, STS measurements of the differential tunnelling conductance (d*I/*d*U*) as a function of the applied bias voltage were performed, reflecting the sample's local density of states. Figure 2a shows single tunnelling spectra measured on the one UC thin FeTe_0.5_Se_0.5_ film and on the Bi_2_Te_1.8_Se_1.2_ substrate at a temperature of *T*=1.1 K. The FeTe_0.5_Se_0.5_ film exhibits an overall U-shaped d*I/*d*U* spectrum with an almost vanishing conductance value at the Fermi level (*E*_F_) and an energy gap of Δ=2.5 meV, defined by half the distance between the two sharp coherence peaks in Fig. 2a. This measured gap value is of the same order of magnitude as measured on the surface of the corresponding superconducting bulk material^23^. In order to determine the critical temperature *T*_c_ of our FeTe_0.5_Se_0.5_ films, we performed temperature-dependent measurements of d*I/*d*U* from 1.1 to 11 K (see Fig. 2b). As expected, the observed energy gap in d*I/*d*U* becomes shallower with increasing temperature and eventually disappears at ∼11 K. The energy gap values for different temperatures (shown in Fig. 2c) were extracted from fits of the background-corrected and symmetrized d*I/*d*U* spectra (for data processing see Supplementary Fig. 3) employing the Dynes density of states, 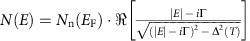, with the density of states in the normal state at the Fermi energy *N*_n_(*E*_F_) and the real part 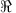, where the parameter *Γ* accounts for the lifetime broadening of the quasiparticles^24^. The fitting of the temperature dependence of the gap in the framework of the Bardeen–Cooper–Schrieffer (BCS) theory^25^ yields an energy gap at 0 K of Δ_0_=2.0 meV and an extracted *T*_c_=11 K, similar to the values that have been extracted from STS on the corresponding superconducting bulk materials^23^,^26^. The temperature dependence of the d*I/*d*U* spectra as well as the derived *T*_c_ value provide strong evidence that the observed gap feature arises from superconductivity in the ultrathin FeTe_0.5_Se_0.5_ film grown on Bi_2_Te_1.8_Se_1.2_.

Now we turn to the investigation of the electronic and magnetic structure of ultrathin FeTe films grown on Bi_2_Te_3_. Figure 2d shows a series of spatially averaged d*I/*d*U* spectra measured at *T*=1.1 K on the Bi_2_Te_3_ substrate as well as on ultrathin FeTe films of different thicknesses. For both embedded UC and top UC FeTe layers a reduced d*I/*d*U* at *E*_F_ with diffuse peaks at energies of ±1 to ±2 meV are found with a gap width and occurrence of the peaks varying with position (Supplementary Fig. 4). A gap of this size and temperature dependence (shown below) has not been observed at the surface of bulk FeTe (refs 27, 28) or for FeTe single layers grown on SrTiO_3_ (ref. 12). The Fermi-level gap gradually vanishes for increased thicknesses of FeTe layers on Bi_2_Te_3_. For example, two UC thin FeTe films show a gap with a significantly reduced depth at *E*_F_. This behaviour suggests that the FeTe/Bi_2_Te_3_ interface plays a significant role for the observed gap structure. In order to reveal the origin of the energy gap in one UC thin FeTe films grown on Bi_2_Te_3_, we thoroughly investigated the temperature and field dependence of the d*I/*d*U* spectra. Figure 2e shows a series of background-corrected and symmetrized temperature-dependent d*I/*d*U* spectra for top UC FeTe on Bi_2_Te_3_. The tunnelling spectrum at *T*=1.1 K exhibits a pair of diffuse peaks at *U*∼2 meV together with a gap region of reduced local density of states at *E*_F_. We observe that the gap depth and peak height in d*I/*d*U* decrease with increasing temperature and finally vanish above *T*=6 K, very reminiscent of a superconducting transition. In order to test this hypothesis, the temperature-dependent spectra were fitted to a Dynes density of states (Fig. 2e). Here we note that a considerable lifetime broadening *Γ* had to be assumed (Supplementary Fig. 5). The temperature dependence of the fitted energy gap values (Fig. 2f) resembles that of a superconducting phase transition (*cf.* Fig. 2c). Kondo correlations, which were shown to cause dip features at *E*_F_ for heavy Fermion systems^29^ and might be present on the Fe lattice as well, are ruled out, as they would show a different temperature dependency of the gap depth (Supplementary Fig. 6). In addition, unlike under-doped cuprates, where a pseudogap phase appears above *T*_c_ (ref. 30), the gap in our tunnelling spectroscopy data always closes at *T*_c_∼6 K, thereby ruling out the presence of a pseudogap state. The spectral features therefore strongly suggest superconducting correlations of the UC FeTe layer. In order to extract the corresponding *T*_c_, we fit the temperature dependence of the gap in the framework of the BCS theory (Fig. 2f), resulting in *T*_c_∼6.5 K. Together with the zero temperature gap value of Δ_0_=1.05 meV extrapolated from the BCS fit we deduce a ratio of 2Δ_0_/*k*_B_*T*_c_∼3.8 for one UC thin FeTe on Bi_2_Te_3_, which is comparable to the value of 4.2 we determine for the FeTe_0.5_Se_0.5_ thin film on Bi_2_Te_1.8_Se_1.2_, but somewhat larger than typical values of 2.5–3.2 for bulk FeTe_1−*x*_Se_*x*_ samples^23^,^26^. We note that the embedded UC of FeTe also shows a similar temperature-dependent behaviour (Supplementary Fig. 7). Our results are consistent with the transport measurements of Fe_1+*x*_Te films grown on Bi_2_Te_3_ (ref. 15) where evidence for superconductivity with a *T*_c_∼12 K was reported. We do not observe any significant effect on the energy gap in a magnetic field up to *B*=2.8 T applied perpendicular to the sample plane (Supplementary Fig. 8). This suggests that the robustness of the pairing with respect to an external magnetic field is similarly strong as for other Fe-based superconducting thin films^7^, and an identical effect has been observed in magnetotransport measurements of a Bi_2_Te_3_/FeTe heterostructure^14^,^15^.

### Spatial variation of superconducting correlation

**Figure 3:**
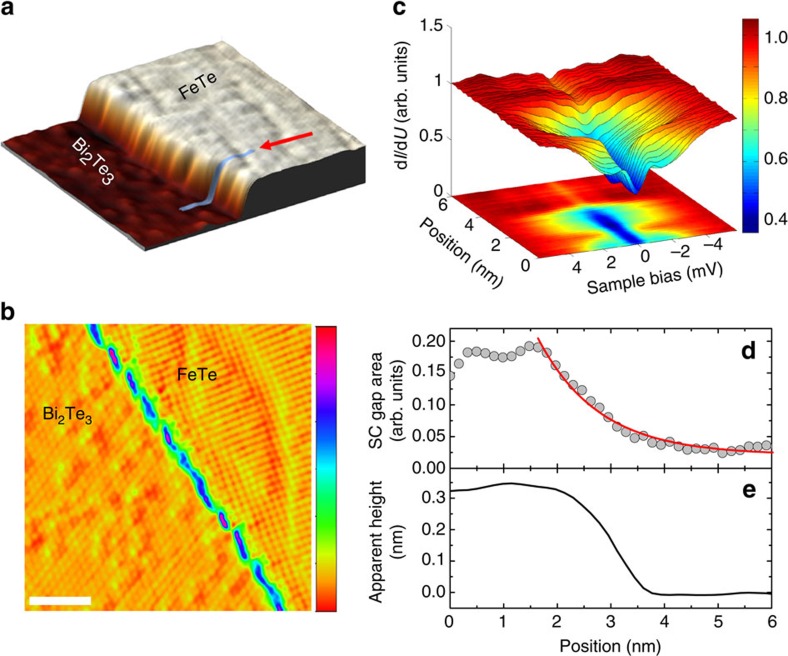
Spatial variation of energy gap across a lateral interface. (**a**) STM topography (25 nm × 25 nm) showing a Bi_2_Te_3_ terrace and an embedded UC thin FeTe layer grown on top (*U*=100 mV, *I*_s_=50 pA). (**b**) Atomically resolved STM topography of a similar area (*U*=50 mV, *I*_s_=50 pA, differentiated with respect to horizontal axis, white scale bar, 3 nm wide). (**c**) Two- and three-dimensional representation of normalized and symmetrized d*I/*d*U* spectra taken across the step along the line indicated in **a** in the direction of the arrow. (**d**) Evolution of the gap (markers), determined from the spectra in **c** by measuring the area enclosed by the gap within a ±1 meV voltage window. The solid line shows a fit to an exponential decay resulting in a decay length of *ξ*=8.9 Å. (**e**) Topographic height taken along the same line used for the data in **c**,**d**.

Having established clear evidence for superconducting correlations in single UC FeTe layers grown on Bi_2_Te_3_ below 6 K, we now focus in more detail on the spatial behaviour of these correlations at the lateral interface of the FeTe layer and the Bi_2_Te_3_ substrate. In particular, we measured the spatial variation of tunnelling spectra across a step edge from the embedded UC FeTe layer to the Bi_2_Te_3_ substrate (Fig. 3a,b). The resulting tunnelling spectra across the interface along the line shown in Fig. 3a obtained at *T*=1.1 K (Fig. 3c) again show the presence of the gap due to superconducting correlations on top of the FeTe layer, which vanishes on the TI substrate. To quantify the decay length of the superconducting correlations in our heterostructure system, we have fitted the spatial dependence of the gap area with an exponential decay function (Fig. 3d), resulting in a decay length of about *ξ*=8.9 Å at *T*=1.1 K. This decay length is a measure for the coherence length of the Cooper pairs^31^,^32^, which is obviously similarly small as, for example, in bulk FeTe_0.6_Se_0.4_ (ref. 26).

### Magnetic properties using SP-STM

Our results on epitaxially grown FeTe layers on Bi_2_Te_3_ confirm that the previously reported two-dimensional superconductivity in FeTe/Bi_2_Te_3_ heterostructures^14^,^15^ is associated with the presence of a superconducting layer of FeTe located at the interface. We now return to the central question of whether the usual bi-collinear AFM order of bulk FeTe (ref. 16) is suppressed in the FeTe layer interfacing Bi_2_Te_3_, as proposed by He *et al*.^14^, or whether it can coexists with the superconducting correlations we observed.

**Figure 4:**
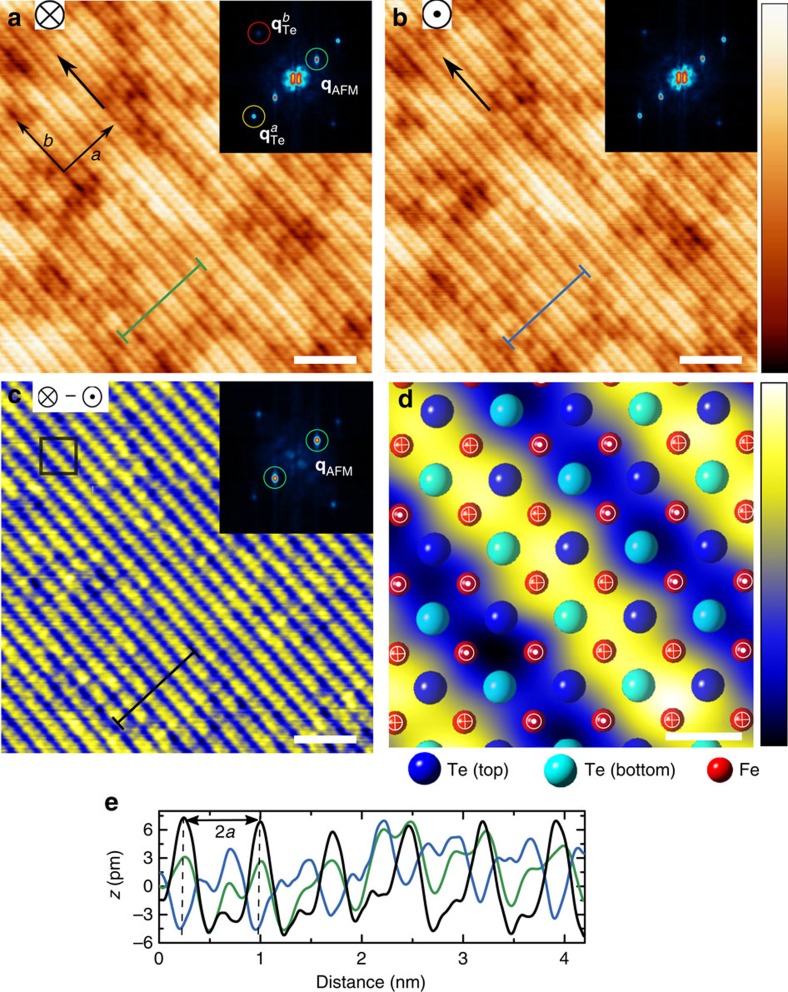
Bi-collinear AFM order in embedded and top UC FeTe. (**a**) SP-STM image recorded at *B*=+1 T showing spin order of one UC FeTe at *T*=1.1 K using an out-of-plane spin-sensitive bulk Cr tip. (**b**) SP-STM image of the same area acquired at *B*=−1 T with an oppositely spin-oriented tip (*U*=300 mV, *I*_s_=220 pA, white scale bars, 3 nm wide, colour scale from 0 to 33 pm apparent height). The arrows in **a**,**b** indicate the same sample spot. The insets in **a**,**b** show FFTs of the corresponding SP-STM images, where the peaks marked by yellow and red circles are associated with the top Te atomic square lattice (Bragg peaks) at **q**_Te_^*a*^ and **q**_Te_^*b*^ (image sizes of all FFTs is 0.55 Å^−1^). Besides the Te Bragg peaks, the FFTs reveal pairs of additional strong peaks (green circles) associated with AFM order at **q**_AFM_=1/2 **q**_Te_^*a*^. (**c**) Spin asymmetry map obtained by subtraction of images **a**,**b**, revealing bi-collinear AFM order in the one UC thin FeTe layer (white scale bar, 3 nm wide). Inset: FFT pattern of the difference image highlighting the peaks in reciprocal space resulting from AFM spin order. (**d**) Model representation of the atomic structure and of the out-of-plane components of magnetic moments superimposed on the zoomed-in spin asymmetry image of **c** (white scale bar, 2.8 Å wide, colour scale from −12 to 12 pm). (**e**) Measured profiles along identical lines drawn in images **a**–**c**, showing the periodicity of AFM order in real space, that is, *λ*=2*a*.

In order to check this assumption, we simultaneously characterized the local superconducting correlations and the atomic-scale spin structure of the ultrathin FeTe films grown on Bi_2_Te_3_ by SP-STS^33^. By using a spin-polarized bulk Cr tip^34^, we excluded the disturbing influence of a local magnetic stray field on the sample while being sensitive to its out-of-plane surface spin component. Figure 4a represents the spin-resolved constant-current image of a single UC thin embedded FeTe layer on Bi_2_Te_3_ as obtained in an external magnetic field of *B*=+1 T applied perpendicular to the sample surface. The SP-STM image shows a clear stripe-like pattern, superimposed on the square atomic lattice of surface Te atoms, which is significantly different from the STM topography obtained with a non-magnetic PtIr tip as presented in Fig. 1f. The Fourier transform (inset of Fig. 4a) of the SP-STM image reveals an additional pair of peaks along the *a* direction with a wave vector **q**_AFM_=1/2 **q**_Te_^*a*^ that is half as long as that of the atomic lattice Bragg peaks. This unidirectional modulation with a characteristic periodicity of *λ*=2*a*=7.5 Å (see Fig. 4e) has been attributed to a direct observation of bi-collinear AFM order of the Fe atoms below the surface of bulk Fe_1+y_Te using SP-STM^16^. To confirm the magnetic origin of the observed superstructure with twice the atomic lattice periodicity, we performed an SP-STM measurement at the same location with an oppositely aligned effective spin at the Cr tip's front end by reversing the magnetic field to *B*=−1 T (see Fig. 4b). Now, the 2*a* superstructure reveals a phase shift (indicated by an arrow) of one atomic lattice unit as expected for a magnetic origin of the contrast. The maximum spin contrast appears between every second Fe lattice site along a diagonal of the surface UC being located between two neighbouring Te sites. This can be explained by the fact that spin-polarized tunnelling primarily results from the 3*d* states of Fe being located below the top Te layer. It is also clearly visible from the line profiles of Fig. 4e along the *a* direction, which were extracted from Fig. 4a,b, and by the 2*a* periodicity along the *a* direction in the out-of-plane spin-polarization distribution of the FeTe layer (Fig. 4c) obtained by subtracting the SP-STM images of Fig. 4a,b. These observations prove that single UC FeTe films on Bi_2_Te_3_ exhibit a bi-collinear AFM structure, similar to the one known for bulk FeTe (ref. 16), but with a non-vanishing out-of-plane surface spin component^35^. Note that the same bi-collinear AFM structure was also observed for the thicker islands in our samples (Supplementary Fig. 9). To our knowledge, this is the first direct real-space observation of magnetic order in an Fe-chalcogenide compound at the single UC thickness limit.

**Figure 5:**
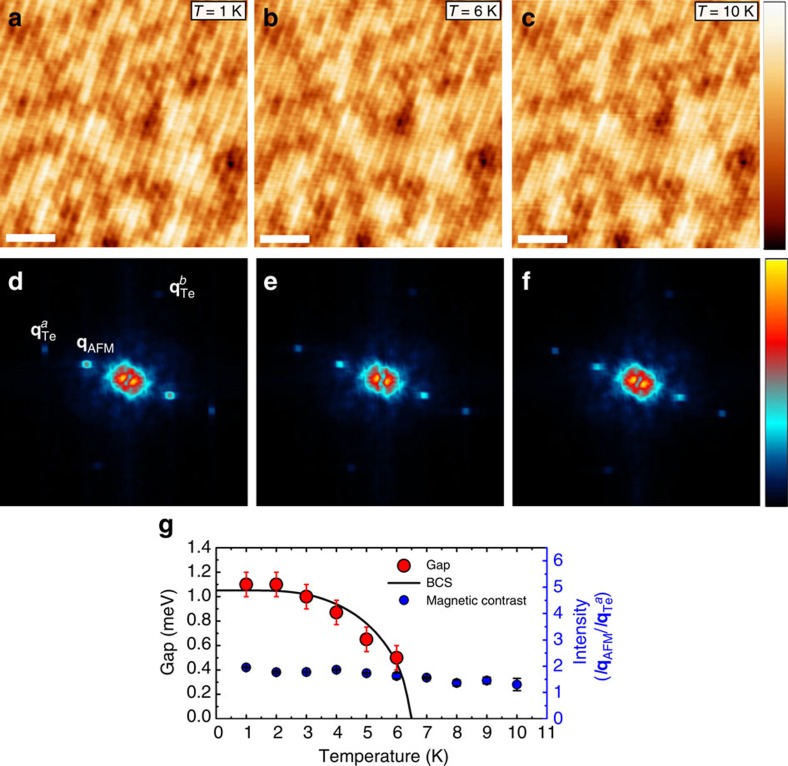
Temperature stability of spin structure for top UC FeTe. (**a**–**c**) SP-STM images (*U*=100 mV and *I*_s_=100 pA, white scale bars, 3 nm wide, colour scale from 0 to 31 pm apparent height) and (**d**–**f**) corresponding FFT data (image size 0.75 Å^−1^) acquired at *B*=0.5 T using an out-of-plane sensitive Cr tip, taken at different temperatures as indicated in **a**–**c**. The position of the surface area is the same in all three SP-STM data sets. The spin structure is robust and the intensity of the magnetic contrast is almost the same at all three temperatures. (**g**) Temperature dependence of the energy gaps Δ plotted together with the magnetic contrast quantified via the ratio of intensities of the FFT spots at **q**_AFM_ and **q**_Te_^*a*^ from the SP-STM images in **d**–**f** and similar images taken at other temperatures. The errors in Δ indicate the maximum range of values used in the Dynes fits, which result in an acceptable agreement with the experimental spectra. The errors in the magnetic contrast indicate the mean deviation as estimated from two data sets.

The observed coexistence of magnetic order in UC FeTe, which is similar to the one found at the surface of the corresponding bulk material^16^, and superconducting correlations, which are not present in bulk FeTe, is quite surprising. In order to find a mutual relationship between magnetic order and superconductivity, we performed SP-STM experiments as a function of temperature from 1 to 10 K. Representative SP-STM images and corresponding FFT data obtained at temperatures *T*=1, 6 and 10 K are shown in Fig. 5a–f. The position of the surface area is the same in all three SP-STM data sets. The spin contrast is clearly reflected by the superstructure with twice the atomic lattice periodicity, and in the corresponding FFT data by the spots at wave vector **q**_AFM_=1/2 **q**_Te_^*a*^. Most notably, the spin structure does not change and the intensity of the spin contrast is almost the same for all three temperatures shown here. Similar SP-STM images at other temperatures have been taken in order to quantify the temperature evolution of the spin contrast via the ratio of FFT intensities of the **q**_AFM_ spots to the Bragg spots at **q**_Te_^*a*^. Figure 5g shows this evolution together with the evolution of the superconducting energy gap taken from Fig. 2f. The comparison clearly demonstrates that the bi-collinear AFM order is largely unaffected by the disappearance of the superconducting correlations. Therefore, our experimental results do not provide any evidence that one kind of order emerges at the expense of the other, nor does the data provide any indication for a microscopic phase separation into regions with superconducting and magnetic order. Our findings for single UC thin FeTe layers on Bi_2_Te_3_ therefore challenge the common belief that optimal superconducting pairing sets in when long-range AFM order is suppressed in the parent compound.

## Discussion

In summary, we have explored the electronic and magnetic structure in a new class of systems, that is, heterostructures consisting of ultrathin Fe-chalcogenide layers of the type FeTe_1−*x*_Se_*x*_ (*x*=0, 0.5) on Bi-based TI substrates. We observe fully developed U-shaped superconducting gaps in FeTe_0.5_Se_0.5_ layers of one UC thickness with a transition temperature of *T*_c_∼11 K, close to the one of the corresponding bulk system (*T*_c_∼14.5 K). For FeTe/Bi_2_Te_3_ heterostructures, our atomic-scale spin-resolved tunnelling spectroscopy measurements provide evidence for the coexistence of superconducting correlations with *T*_c_∼6.5 K and bi-collinear AFM order in the one UC FeTe films. Although the coexistence of static magnetic order and superconductivity was observed earlier by spatially averaging techniques in several systems such as Fe-pnictides^36^,^37^, heavy fermion compounds^38^ and Fe-chalcogenides^39^, the exact microscopic picture of the coexistence still remained unclear because of a lack of spatially resolved data^40^. Here we can compare the wavelength of the AFM order of *λ*=2*a*=7.5 Å with the size of the Cooper pairs, inferred from the coherence length, of *ξ*=8.9 Å, giving *ξ*/*λ*∼1.2. The electron distance in the pairs is rather small, which is typical for Fe-based SCs, and apparently just large enough such that the effective Zeeman field induced by the AFM order cancels out along the length of the Cooper pair. The relative sizes of *λ* and *ξ* therefore might be crucial for the coexistence of pairing and long-range AFM order in this material. Our surprising findings may stimulate further theoretical studies on the relationship between superconductivity and AFM order in Fe-based SCs.

We finally note that the leakage of the gap into the TI substrate across the FeTe–Bi_2_Te_3_ interface (Fig. 3c) indicates the presence of superconducting correlations in the TI material close to the interface. The atomic sharpness of this interface suggests that the topological surface state of the TI substrate stays intact as also shown recently by photoemission experiments for the case of a FeSe–Bi_2_Se_3_ heterostructure^41^. The FeTe–Bi_2_Te_3_ interface therefore provides an ideal platform to study the interesting physics of Dirac fermions interacting with Cooper pairs.

## Methods

### Samples

Bulk TI single crystals of Bi_2_Te_3_ and Bi_2_Te_1.8_Se_1.2_ used in this study as substrates were synthesized using a Stockbarger method and have been well characterized using angle-resolved photoemission spectroscopy, powder X-ray diffraction, inductively coupled plasma atomic-emission spectroscopy and potential Seebeck microprobe measurements^21^,^42^. For all substrates the Dirac point is located energetically below the Fermi level indicating *n* doping. Fe-chalcogenide film preparation and characterization were carried out in a UHV system with a base pressure below 1 × 10^−10^ mbar. The TI crystals were cleaved *in situ* under UHV conditions and high-quality FeTe_1−*x*_Se_*x*_ (*x*=0, 0.5) thin films were prepared by depositing 0.5–1 ML Fe at 300 K on-top of Bi_2_Te_3_ and Bi_2_Te_1.8_Se_1.2_ substrates, respectively, using molecular beam epitaxy, followed by annealing up to a maximum temperature of 315 °C for 15 min. Fe deposited on Bi_2_Te_3_ reacts with the substrate upon annealing, most likely via a substitutional process of Bi by Fe (ref. 43), leading to a high-quality defect-free FeTe film.

### Experimental techniques

All STM/STS experiments were performed with an STM in UHV at temperatures between 1.1 and 14 K (ref. 24). A magnetic field *B* of up to 3 T can be applied perpendicular to the sample surface. Topography images were obtained in constant-current mode with stabilization current *I*_s_ and bias voltage *U* applied to the sample. STS data were obtained using a lock-in technique to record the differential tunnelling conductance d*I*/d*U* by adding an AC modulation voltage *U*_mod_ (given in r.m.s.) to the bias voltage, after stabilizing the tip at *I*_s_ and *U*, switching off the feedback, and ramping the applied bias *U*. We used cut PtIr or electrochemically etched W tips (both *in situ* flashed) for spin-averaged imaging and spectroscopy measurements. For spin-resolved measurements, we used bulk Cr tips, which were prepared by electrochemical etching followed by a high-voltage field emission treatment using W(110) or Ta(001) as a substrate.

### Data availability

The authors declare that the main data supporting the findings of this study are available within the article and its Supplementary Information files. Extra data are available from the corresponding author upon request.

## Additional information

**How to cite this article**: Manna, S. *et al*. Interfacial superconductivity in a bi-collinear antiferromagnetically ordered FeTe monolayer on a topological insulator. *Nat. Commun.* **8**, 14074 doi: 10.1038/ncomms14074 (2017).

**Publisher's note**: Springer Nature remains neutral with regard to jurisdictional claims in published maps and institutional affiliations.

## Supplementary Material

Supplementary Information Supplementary Figures 1-9 and Supplementary References
