# Supplementary material for: Interfacial superconductivity in a bi-collinear antiferromagnetically ordered FeTe monolayer on a topological insulator
Source: Nat Commun. 2017 Jan 17;8:14074. doi: 10.1038/ncomms14074 (PMC5247605; doi:10.1038/ncomms14074)
Supplement: Supplementary Information — Supplementary Figures 1-9 and Supplementary References [file ncomms14074-s1.pdf]

Supplementary Figures:

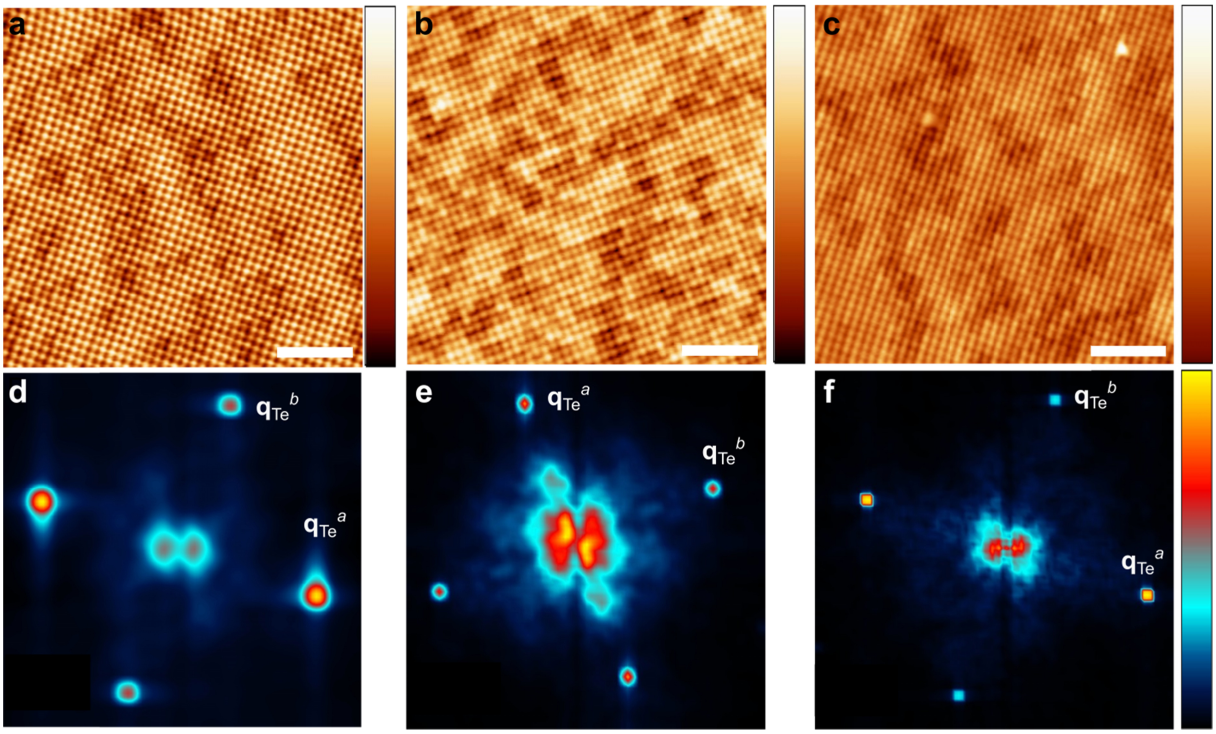

Supplementary Figure 1 | STM topographs and FFTs of FeTe films of various thicknesses. (**a**-**c**) STM topographs of FeTe films of different thicknesses grown on Bi_2Te_3 (white scale bars are 3 nm wide, **a**: embedded UC, U = 100 mV, I_s = 100 pA, color scale from 0 to 104 pm apparent height; **b**: embedded + top UC, U = -15 mV, I_s = 300 pA, color scale from 0 to 55 pm apparent height; **c**: thick layer, U = 280 mV, I_s = 100 pA, color scale from 0 to 66 pm apparent height). (**d**-**f**) Corresponding FFT images of **a**-**c** (image sizes are 0.65 Å^{-1}). The ratios of the lattice constants in *a*- and *b*-direction, *a*/*b*, derived from the atomically resolved topographs and their FFTs are: (**a**,**d**) $a/b \sim 1.05$, (**b**,**e**) $a/b \sim 1.01$, (**c**,**f**) $a/b \sim 1.07$.

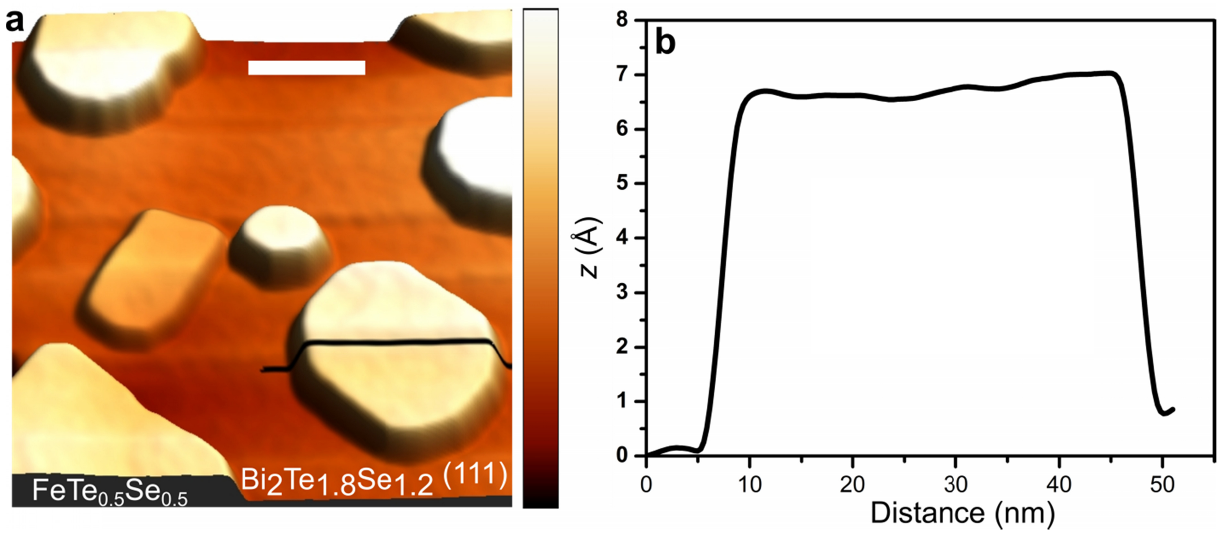

Supplementary Figure 2 | Thin film of $FeTe_{0.5}Se_{0.5}$ grown on $Bi_2Te_{1.8}Se_{1.2}$. (**a**) STM topograph showing one embedded UC thick island as well as several top UC thick islands (U = 100 mV, I_s = 150 pA, white scale bar is 21 nm wide, color scale from 0 to 1.3 nm apparent height). (**b**) Height profile of one of the top UC islands along the line indicated in **a**.

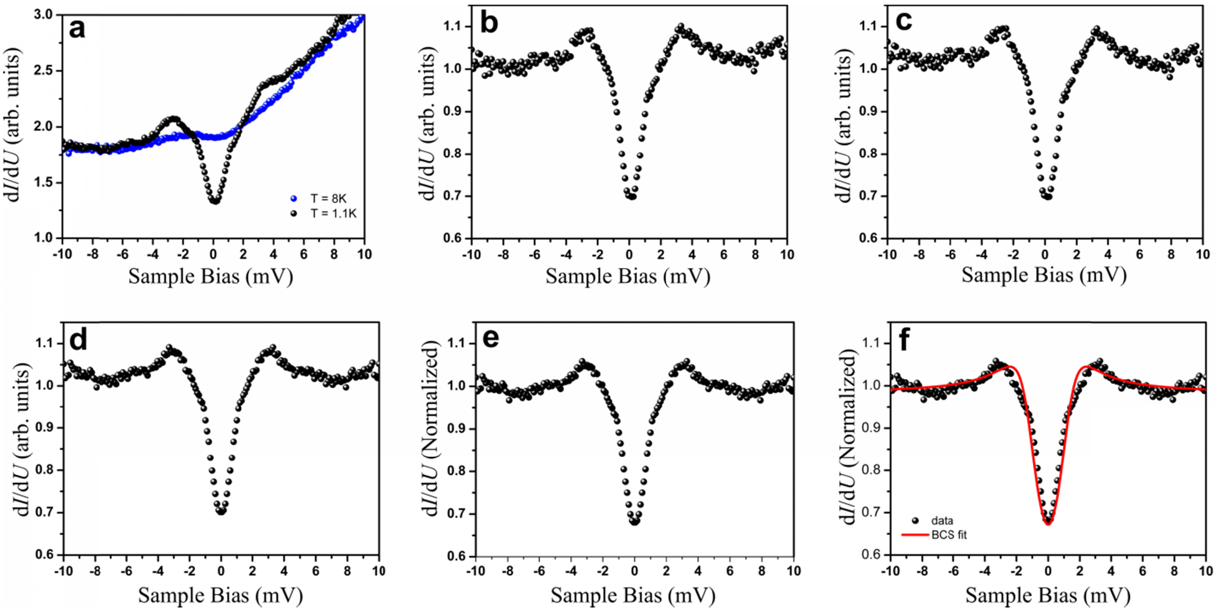

Supplementary Figure 3 | Description of the dI/dU data processing used for fitting. (**a**) Raw data of dI/dU spectra measured on a top UC FeTe layer at temperatures of T = 1.1 K, i.e. below the critical temperature T_c, and at T = 8 K, i.e. above T_c, as indicated (Measurement parameters: U = 10 mV, I_s = 200 pA, U_{mod} = 0.15 mV). (**b**) Background correction: the spectrum taken at T = 1.1 K was divided by the spectrum measured at T = 8 K in order to correct the non-constant background sample density of states in the normal state. (**c**) Slope correction: a constant slope has been subtracted in order to have dI/dU(-10mV) = dI/dU(+10mV). (**d**) Symmetrization with respect to zero bias: average of the original spectrum from **c** and its mirror with respect to zero bias. (**e**) Normalization: division by dI/dU(±10mV). (**f**) Fit of the measured spectrum (dots) to BCS theory (red line) using the Dynes function $N(E) = N_\mathrm{n}(E_\mathrm{F}) \cdot \Re\left[\frac{|E|+i\Gamma(T)}{\sqrt{\left(|E|+i\Gamma(T)\right)^2-\Delta^2(T)}}\right]$ for the density of states $N(E)$ of the sample as described in [1]. Here, $N_\mathrm{n}(E_\mathrm{F})$ is the sample density of states in the normal state which is assumed to be energy independent after normalization, $\Re$ denotes the real part, $\Gamma(T)$ is the resulting temperature dependent quasiparticle lifetime broadening factor, and $\Delta(T)$ is the resulting temperature dependent energy gap.

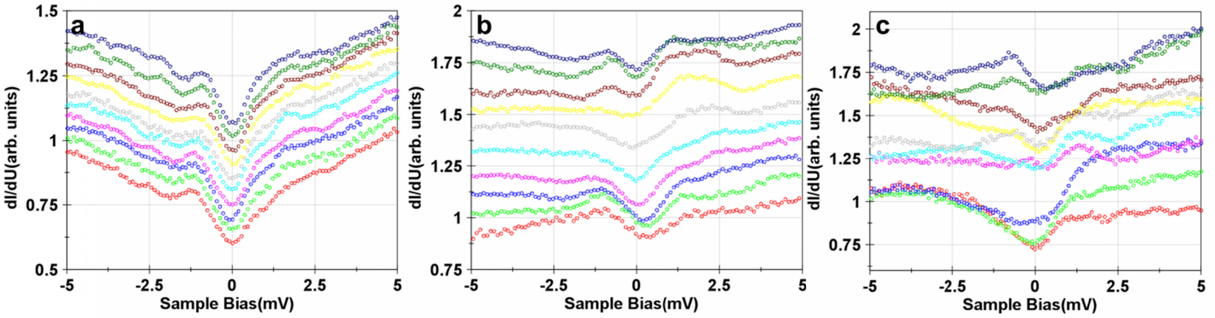

Supplementary Figure 4 | Spatial variation of d*I*/d*U* spectra. (**a**) Spectra measured at $T = 1.1$ K on the embedded UC FeTe ($U = 6$ mV, $I_s = 300$ pA, $U_{mod} = 0.2$ mV). The spectra have been taken on 10 points along a line of 1 nm length. (**b**) Spectra measured at $T = 1.1$ K on the top UC FeTe ($U = 10$ mV, $I_s = 600$ pA, $U_{mod} = 0.25$ mV). The spectra have been taken on 10 points along a line of 2 nm length. (**c**) Spectra measured at $T = 1.1$ K on the 2 UC thick FeTe ($U = 5$ mV, $I_s = 300$ pA, $U_{mod} = 0.2$ mV). The spectra have been taken on 10 points along a line of 4 nm length. All spectra have been normalized to the average of the d*I*/d*U* values at +5 mV and -5 mV, and are vertically shifted for clarity.

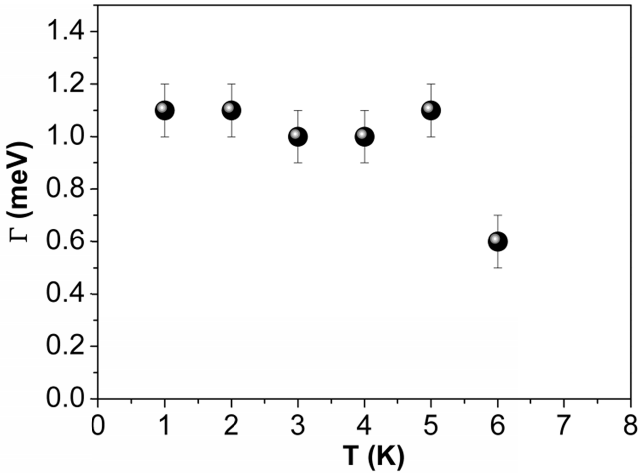

Supplementary Figure 5 | Temperature dependence of the lifetime broadening factor. Lifetime broadening factor Γ resulting from the fitting of the spectra measured on a top UC FeTe thin film as shown in Fig. 2e of the main manuscript. The errors indicate the maximum range of values of Γ in the Dynes function using BCS theory which result in an acceptable fitting of the experimental spectra.

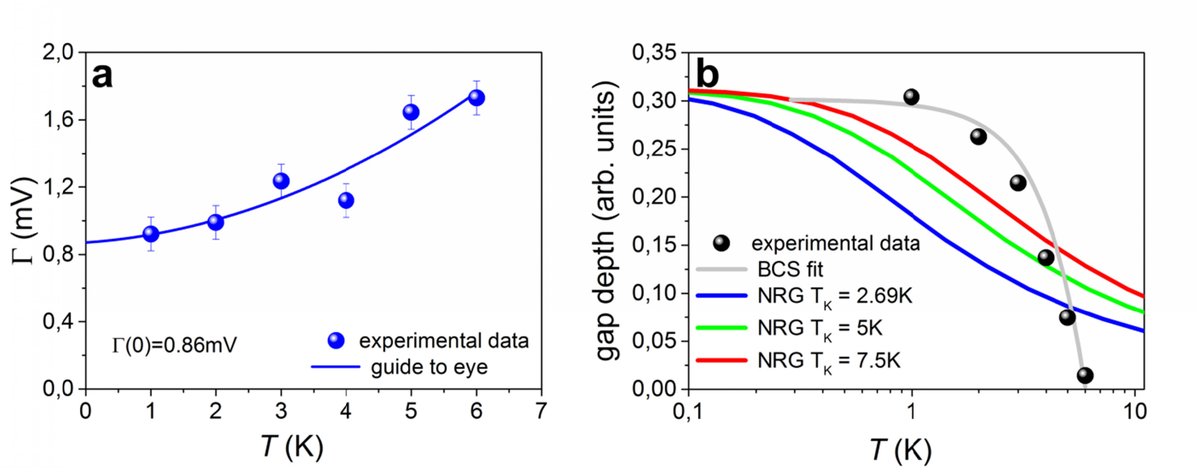

Supplementary Figure 6 | Comparison of Kondo model and BCS theory analysis. In order to rule out, that the spectroscopic gap-feature at zero bias, which was observed for the thin FeTe films on Bi_2Te_3, is due to a Kondo lattice behavior of the Fe atoms [2], the data of Fig. 2e of the main manuscript has been analyzed within a Kondo model. (**a**) The d*I*/d*U* spectra in Fig. 2e (before background correction and symmetrization) have been fitted to Fano-functions $\frac{dI}{dU}(U) \propto c_1 + c_2\frac{(q+\varepsilon)^2}{1+\varepsilon^2}$, with $\varepsilon = \frac{U-U_K}{\Gamma(T)}$ [3]. Here, c_1 and c_2 are offsets and amplitudes of the spectra, respectively, q is the so-called form factor, U_K is the voltage position, and $\Gamma(T)$ is the half-width at half maximum of the Kondo resonance. The resulting temperature dependence of Γ is shown by the markers in **a**. The errors indicate the maximum range of values for Γ in the Fano-functions which result in an acceptable fitting of the experimental spectra. Γ interpolates to a value of $\Gamma(0) \approx 0.86$ mV, as shown by the line as a guide to the eye, which would correspond to a Kondo temperature of $T_\mathrm{K} = \frac{0.27\Gamma(0)}{k_\mathrm{B}} \approx 2.69\,\mathrm{K}$, using Wilson's definition of the Kondo temperature [4]. (**b**) The temperature evolution of the extracted gap depth of the experimental data (markers) is compared to NRG calculations for a spin-1/2 Kondo impurity in the strong coupling regime [5] assuming different Kondo temperatures (colored lines). Obviously, the temperature dependence of the gap depth does not fit to that of a Kondo lattice, independent of the choice of T_K. In contrast, it fits well to the temperature dependence of the gap depth of a superconductor extracted from the BCS model (grey curve). The depth values at different temperatures for the grey curve were obtained employing a Dynes density of states with Δ as calculated from BCS theory [1,6].

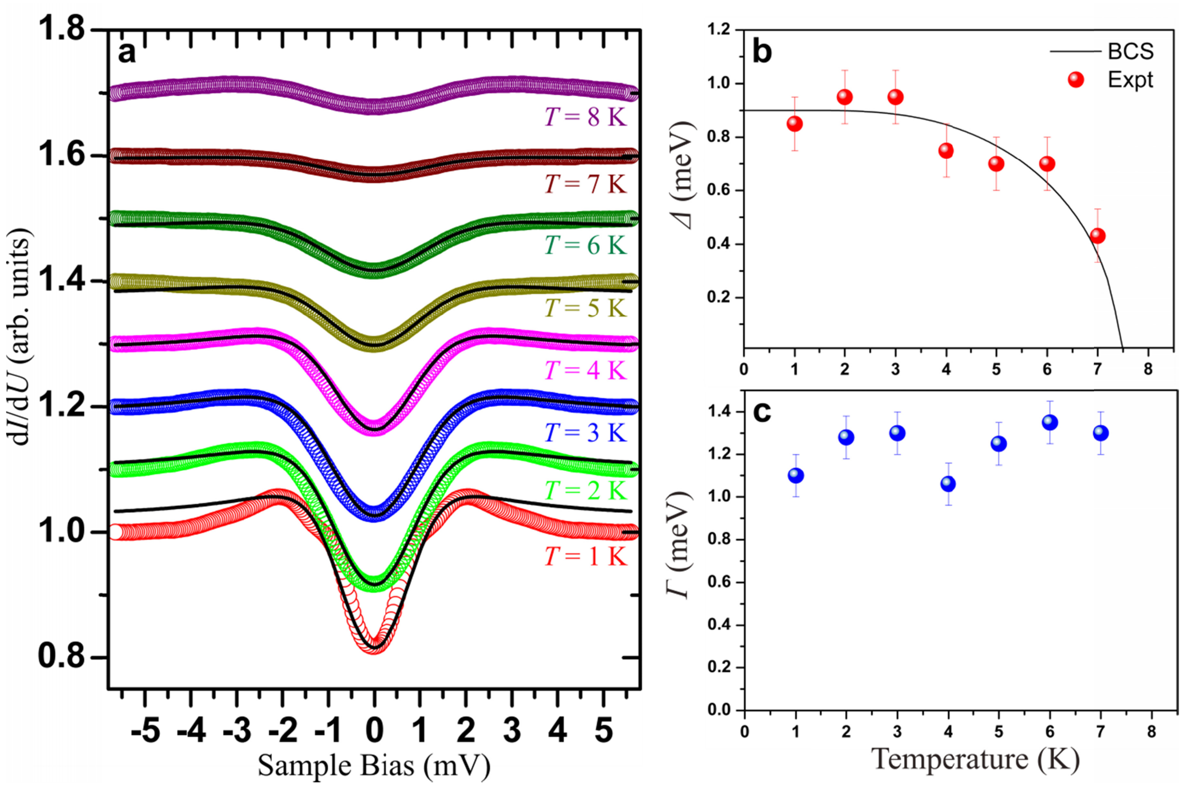

Supplementary Figure 7 | Gap temperature dependence of embedded UC. (**a**) Background corrected (using the data measured at T = 10 K) and symmetrized dI/dU spectra (colored markers) measured at different temperatures as indicated. The spectra are spatially averaged over an area of 1 nm^2. Measurement parameters: U = 6 mV, I_s = 300 pA, U_{mod} = 0.2 mV. The lines show fits to BCS theory using the Dynes function. (**b**) Temperature dependence of the gap values resulting from the fits in **a** (markers). The line shows the fit to the temperature dependence expected from BCS theory resulting in $\Delta(0) = 0.9$ meV and T_c = 7.5 K. (**c**) Lifetime broadening factors resulting from the fits in **a**. The errors in **b** and **c** indicate the maximum ranges of values of Δ and Γ, respectively, in the Dynes function using BCS theory which result in an acceptable fitting of the experimental spectra.

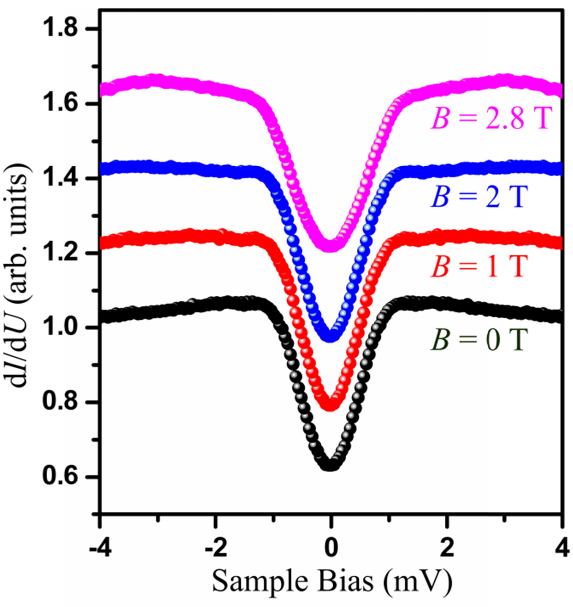

Supplementary Figure 8 | Magnetic field dependence of gap. Differential tunneling conductance spectra measured at T = 1.1 K on the embedded UC, in various magnetic fields B applied perpendicular to the sample surface as indicated. Measurement parameters: U = 6 mV, I_s = 300 pA, U_{mod} = 0.2 mV. The data is background corrected and symmetrized. There is basically no change up to B = 2 T, and only a slight broadening of the gap at B = 2.8 T.

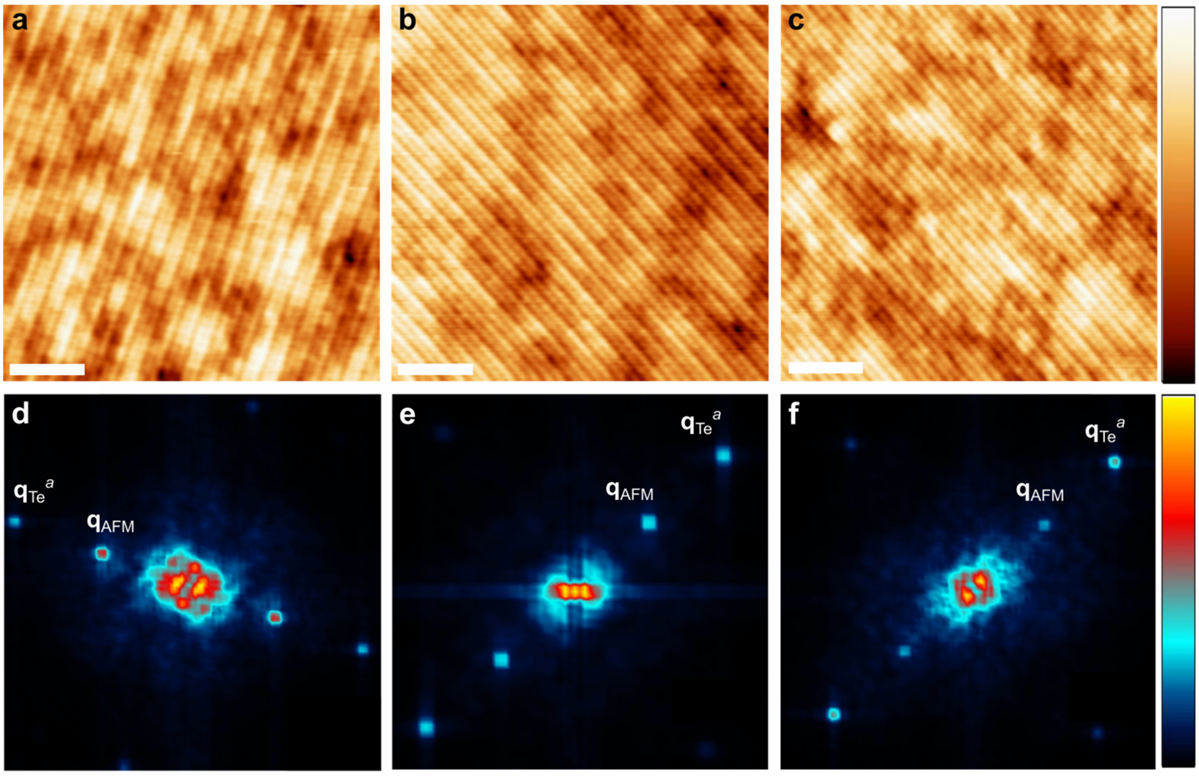

Supplementary Figure 9 | SP-STM of FeTe films of different thicknesses. (**a-c**) Spin-resolved STM images of FeTe films of various thicknesses grown on Bi_2Te_3 taken at $T = 1.1$ K. All white scale bars are 3 nm wide. The color scale is from 0 to 65 pm apparent height. (**a**) Top UC FeTe, $U = 100$ mV, $I_s = 100$ pA, $B = 0.5$ T; (**b**) Embedded + Top UC FeTe, $U = 200$ mV, $I_s = 100$ pA, $B = 1$ T; (**c**) Thick FeTe, $U = 70$ mV, $I_s = 100$ pA, $B = 1$ T. (**d-f**) FFT images corresponding to **a-c** (image sizes are 0.54 Å^{-1}). The spin contrast is visible in the topographs by a superstructure with twice the atomic lattice periodicity, and in the corresponding FFT images by the spots at wave vector $\mathbf{q}_{AFM} = 1/2\ \mathbf{q}_{Te}^{a}$. The intensity of the spin contrast is comparable for all three thicknesses.

Supplementary References:

1. Löptien, P., Zhou, L., Khajetoorians, A. A., Wiebe, J. & Wiesendanger, R. Superconductivity of lanthanum revisited: enhanced critical temperature in the clean limit. *J. Phys.: Condens. Matter* **26**, 425703 (2014).
2. Ernst, S. *et al.* Emerging local Kondo screening and spatial coherence in the heavy-fermion metal YbRh2Si2. *Nature* **474**, 362-366 (2011).
3. Ternes, M., Heinrich, A. J. & Schneider, W.-D. Spectroscopic manifestations of the Kondo effect on single adatoms. *J. Phys.: Condens. Matter* **21**, 053001 (2009).
4. Khajetoorians, A. A. *et al.* Tuning emergent magnetism in a Hund's impurity. *Nature Nanotechn.* **10**, 958-965 (2015).
5. Costi, T. A. Kondo effect in a magnetic field and the magnetoresistivity of Kondo alloys. *Phys. Rev. Lett.* **85**, 1504-1507 (2000).
6. Tinkham, M.. Introduction to superconductivity: second edition (*Dover Books on Physics*) (Vol i) (2004).
